# A novel resilience analysis methodology for airport networks system from the perspective of different epidemic prevention and control policy responses

**DOI:** 10.1371/journal.pone.0281950

**Published:** 2023-02-27

**Authors:** Jiuxia Guo, Zongxin Yang, Qingwei Zhong, Xiaoqian Sun, Yinhai Wang

**Affiliations:** 1 College of Air Traffic Management, Civil Aviation Flight University of China, Guanghan, Sichuan, China; 2 National Key Laboratory of CNS/ATM, School of Electronic and Information Engineering, Beihang University, Beijing, China; 3 Department of Civil and Environmental, University of Washington, Seattle, WA, United States of America; Al Mansour University College-Baghdad-Iraq, IRAQ

## Abstract

As the COVID-19 pandemic fades, the aviation industry is entering a fast recovery period. To analyze airport networks’ post-pandemic resilience during the recovery process, this paper proposes a Comprehensive Resilience Assessment (CRA) model approach using the airport networks of China, Europe, and the U.S.A as case studies. The impact of COVID-19 on the networks is analyzed after populating the models of these networks with real air traffic data. The results suggest that the pandemic has caused damage to all three networks, although the damages to the network structures of Europe and the U.S.A are more severe than the damage in China. The analysis suggests that China, as the airport network with less network performance change, has a more stable level of resilience. The analysis also shows that the different levels of stringency policy in prevention and control measures during the epidemic directly affected the recovery rate of the network. This paper provides new insights into the impact of the pandemic on airport network resilience.

## 1. Introduction

In different countries and regions, COVID-19 resulted in a significant decline of travel frequencies and distances, and a plummeting number of transportation users [1]. According to International Civil Aviation Organization (ICAO) statistics, the number of global aviation passengers (including both international and domestic travel) fell by 60% from 2019 to 2020. According to Airports Council International (ACI) statistics, global airport revenue losses reached US$125 billion [2]. The influence of COVID-19 on the aviation industry was striking. COVID-19 was controlled by closing firms and cities, increasing social distance, and limiting travel [3]. These measures had ambiguous effects. On the one hand, they forcefully controlled COVID-19. On the other hand, they did considerable economic damage, and remained significant impediments to aviation’s resurgence. According to the ICAO data, compared to 2019, the world passenger traffic was decreased by 60% in 2020, by 49% in 2021, and by 26% to 28% in 2022 [4]. Different countries/regions adopted different epidemic prevention and control measures, which had different impacts on airport networks. For example, the Chinese government adopted a traffic control policy that restricted the movement of people and vehicles, as well as a home lockdown policy that required people must not to go out except for buying daily necessities or in emergency situations. In contrast, the United States and European governments recommended movement restriction and reduction of transport volume, and recommended not leaving the house [5]. In light of these differences in public health measures, it is critical to examine the differential impacts of COVID-19 on airport systems, in order to anticipate the recovery process, and better prepare for future incidents [6].

To the best of our knowledge, a model combining performance and resilience assessment with the recovery of different regional airport networks (with their different levels of stringency policy of prevention and control measures in the post-epidemic period) has not been studied in the literature. Accordingly, we address the following three problems:

1. How to develop and suggest a model to quantitatively evaluate the resilience and recovery of aviation network due to an epidemic.
2. Which airport operational factors should be considered to evaluate resilience, such as actually airport network performance changes, in light of different levels of stringency policy of prevention and control measures?
3. What is the impact of epidemic prevention and control on network recovery rates?

This paper considers the Chinese airport network (CAN), European airport network (EAA) and the USA airport network (AAN) as examples in a comprehensive method to analyze each airport network’s performance, and explores the impact of factors such as different levels of stringency policy of prevention and control measures on the recovery time and resilience level of the aviation network. We selected a stringency index which considered the thirteen metrics (e.g. school closures; workplace closures; cancellation of public events, etc.) as the criteria to assess the stringency policy. Because ICAO predicted a V-shaped or U-shaped expected recovery curve for the aviation industry when the COVID-19 broke out [7], this paper adopts monotonic recovery functions.

The remainder of this paper is outlined as follows. In Section 2, the related literature is reviewed. In Section 3, the paper proposes a CRA method and analyzes airport networks recovery with performance loss and different prevention and control measures policies. In Section 4, we compared the changes of different airport networks performance and resilience. Section 5 summarizes our findings, addresses the limitations of the model, and proposes new paths for future research.

## 2. Literature review

To highlight this study’s positioning and contribution, we review the relevant literature on complex network resilience and recovery.

### (1) Complex networks

Understanding the structure and dynamics of complex social technology systems with social connectivity attributes has been made easier using network methodology. ZhangJ2010Zhang et al. (2010) used complex network theory to analyze aviation networks [8]. Their research results demonstrated the applicability of the complex network theory to the analysis of network topology, and the impact of unexpected events, such as disease outbreaks, on the performance of airport networks. Wandelt et al. (2019) reviewed and compared the evolution of domestic airport networks in eight countries between 2002 and 2013 and found that all the networks had experienced significant growth in passenger traffic and exhibited small-world characteristics [9]. ZengxiaozhouZeng used network efficiency, cluster coefficients, degree distribution, and connectivity coefficients to assess the performance of airport networks [10]. Holme et al. (2002) assessed network performance using the geodetic length between nodes [11]. 单击或点击此处输入文字。Huang et al. (2011) combined inter-node flow with path length to construct a two-dimensional complex network characteristic index [12]. XingPPan et al. (2018) proposed a stress-strength-balance weighted network method to evaluate system loss, by considering the changes in the trends of specific indicators [13]. Therefore, complex networks are often used to analyze network structure characteristics of airports and other transportation systems.

### (2) Complex network resilience

Improving the robustness of transportation networks is an important challenge, and in recent years, researchers have increasingly studied the resilience of transportation systems from the perspective of complex networks [14]Wandelt_s_2021. Complex network resilience the ability to retain performance during and after disruptions and resume a normal state of operation promptly after disturbances [15]. PatriarcaRPatriarca et al. (2018) suggested that studies on resilience should change their focus from defined resilience to modeling from a safety perspective [16]. Similar sentiments have encouraged scholars to incorporate resilience into the aviation field. Filippone et al. (2016) developed a model to assess the resilience of air traffic management (ATM), defining ATM resilience management by identifying the most efficient task assignment [17]. Lordan et al. (2014) used the complex network theory to assess the robustness of airport networks from the viewpoints of the government and airport companies [18]. Zeng (2012) used complex networks to investigate the survivability of CAN in response to government policies, and discovered that attacking a large hub airport had a significant influence on the efficiency of the airport network [10]. Because robustness is an amorphous metric, Janic et al. (2013) proposed a model to quantify the resilience of the transportation network of 16 U.S. airports using Hurricane Sandy as an impact event [19]. Guo et al. (2021) established an airport network resilience model to assess CAN resilience performance during public health events [20]. Bauranov et al. (2021) constructed the AAN resilience model and measured the AAN’s various network characteristics and resilience under COVID-19 based on network characteristic indicators and carbon dioxide emissions [21].

### (3) Complex network recovery

A network’s resilience is influenced by various factors such as network structure, performance loss and recovery time etc. After discussing the factors influencing resilience in chemical processes and their importance, Dinh et al. (2012) proposed that future resilience evaluation models should be multi-level and multi-attribute [22]. To quantify the resilience system’s recovery process, Wuellner et al. (2010) used airport company data to construct the AAN, and assessed the geographical network structure with spatial cost and analyzed network resilience and recovery after an attack with both “diamond” and “chain” [23]. Shortly thereafter, Henry argued that resilience research should include recovery time [24]. However, no study has explored the impact of global emergencies on airport networks, or the recovery of airport networks after such events. Regarding aviation network recovery after COVID-19, many studies have explored recovery patterns using ARIMAX models [25], a triple exponential smoothing model [26], and different ranges of recovery functions, such as V-shaped, L-shaped, and W-shaped. Both, functions recover slowly in the early stage and rapidly or gradually recover in the later stage [27]. Domaneschi et al. (2016) proposed a recovery index based on time and system characteristics to analyze the overall system, and a general recovery function that can be applied to a variety of recovery modes [28]. In 2020, the performance of the aviation network was significantly reduced; therefore, studies on the shape of the system recovery function for the post-epidemic period have used a U-shape or L-shape [29]SUN_x_2021_airports. Kou (2019) proposed a post-earthquake bridge recovery model based on sigmoid functions, which can be obtained in various functional forms such as positive and negative exponentials by adjusting the coefficients [30]. A negative exponential function indicates sufficient resources for recovery, and the system can recover to a better functional level within a short period. A positive exponential function indicates either that the system components are damaged, or that there are insufficient resources for recovery, and that the system will take a long time to reach a better functional level.

Therefore, we propose a CRA model to study the correlation between network resilience and epidemic prevention and control policy responses for CAN, EAN, and AAN, using real air traffic data. The process includes assessing airport network performance using two-dimensional complex network characteristic index after COVID-19, and analyzing airport resilience under different policies based on multi-factor recovery model.

## 3. Methodology

The data used in this study were obtained from VarFlight (https://map.variflight.com/), which includes 232 airports in mainland China, 82 airports in Europe, and the top-110 passenger airports in the USA. In addition, we obtained the total passenger and flight data of Chinese, European and U.S. airports for 2019 and 2020. Passenger data regarding Chinese airports were obtained from the Civil Aviation Administration of China (http://www.caac.gov.cn/), European airports from the EUROCONTROL website (https://www.eurocontrol.int/), and United States airports from the Federal Aviation Administration (https://www.faa.gov/).

### 3.1. Airport network model

In the study, we construct an airport network model based on complex network and graph theory. The airport network graph (*G*) comprises a set of nodes *V* and edges *E*, denoted as *G* = (*V*,*E*) [31]. The adjacency matrix *A* of the airport network is represented by the edges between the airport nodes, where *A* = {*a*_*ij*_} is given by Eq 1.


$$A=\{\begin{array}{l} a_{ij}=1;\;i\;is\;connected\;to\;j\\ a_{ij}=0;\;i\;is\;not\;connected\;to\;j \end{array}$$ (1)

Statistical indicators of complex networks include the degree of nodes, cluster coefficients (clustering coefficients), average path lengths, efficiency, degree correlation, and cluster degree correlations [32–36].

Following the literature, we selected the network topology indicators in complex network theory to assess the topology structure of the airport network, such as the average degree ($\bar{k}$), average clustering coefficient ($\bar{C}$), efficiency *(E_ff_)* and average path length ($\bar{L}$). The proposed airport network performance indicators (*R*_*Performance*_) and recovery indicators are used to analyze the robustness and resilience of each airport network.

### 3.2. CRA model

To evaluate the resilience ability of complex airport networks during COVID-19, we propose a CRA model that considers the performance indicator and integrated recovery indicator.

#### 3.2.1 Performance indicators

In an airport network system, the connectivity between airport nodes directly affects the network topology structure, where the throughput at airports affects the network performance. We construct performance indicators for airport networks using the passenger throughput of out-degree and in-degree, which is defined in Eq 2:

$$R_{Performance}=\frac{1}{N(N-1)}\sum_{i,j\in G;i\neq j}^{N}\frac{1}{d_{ij}}(g_{ij}+g_{ji})$$ (2)

Here, *d*_*ij*_ is the length of the path between airport *i* and *j*, *g*_*ij*_ is the number of passengers between *i* and *j*, *g*_*ji*_ is the number of passengers between airport *j* and *i*, and *N* is the number of airports in airport network.

The performance loss of the airport network can be defined as shown in Eq 3:

$$R_{loss}=R_{Performance}^{'}-R_{Performance}^{''}$$ (3)

where *R*_*damage*_ is an airport network loss indicator, $R_{Performance}^{'}$ and $R_{Performance}^{''}$ are the pre- and post-event airport network performances, respectively.

#### 3.2.2. Integrated recovery indicators

**Fig 1:**
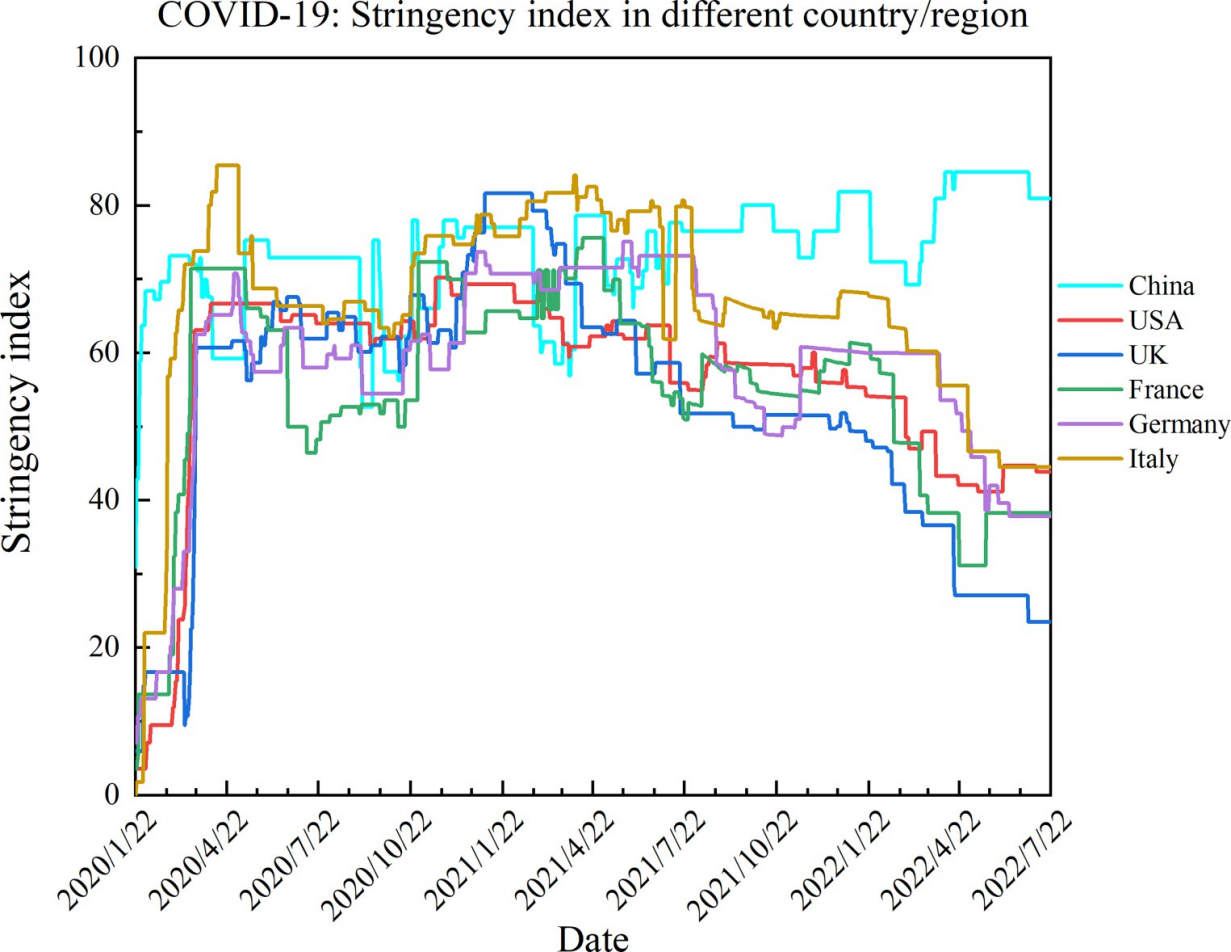
COVID-19: Stringency index in different countries.

Different epidemic prevention and control measures would lead to different levels of confirmed cases and deaths, and some countries and territories in the world failed to maintain their prevention and control, causing the COVID-19 pandemic to rebound in even higher waves [37]. Therefore, we assessed the different strengths of prevention and control policy responses in each country and region using the stringency index. The stringency index was the government’s response to epidemic prevention and control measures which considered 13 indicators: school closures, workplace closures, cancellation of public events, restrictions on public gatherings, closures of public transport, stay-at-home requirements, public information campaigns, restrictions on internal movements, international travel controls, testing policy, extent of contact tracing, face covering, and vaccine policy [5]. In Fig 1, the higher score of the stringency index reflects a stricter response, each taking a value between 0 and 100.

Fig 1 shows the response to the epidemic prevention and control measures taken by six countries from January 2020 to July 2022. Each country had taken stricter prevention and control measures in the first three months, but after July 2021, the severity of each country was different. However, only the Chinese government has continued to adopt severe prevention and control policy.

Accordingly, we developed a comprehensive recovery model that considers recovery measures and epidemic prevention and control measures, which is defined in Eq 4:

$$R_{re\mathrm{cov}ery}=R_{Performance}(1-\frac{t}{T})\frac{1}{1+e^{-\alpha (t-\frac{T}{2})}}$$ (4)

where *R*_recovery_ is an airport network system recovery indicator, *a* is a composite recovery index that indicates the recovery trend under different epidemic prevention and control measures, *t* is the recovery time (months), and *T* is the maximum acceptable recovery time (months) declare by airport administration. Here, we assume that the ICAO-predicted post-pandemic recovery time for civil aviation is the maximum acceptable recovery time for national flights [2].

The recoverability evaluation model was a penalty function type, and the system network recoverability decreased as the recovery time continued. If the recovery time is greater than the maximum recovery time declared by the airport administration ($1-\frac{1}{T}<0$), the system network resilience recovery is feasible; that is, without aid, the network will not return to its original performance level.

**Fig 2:**
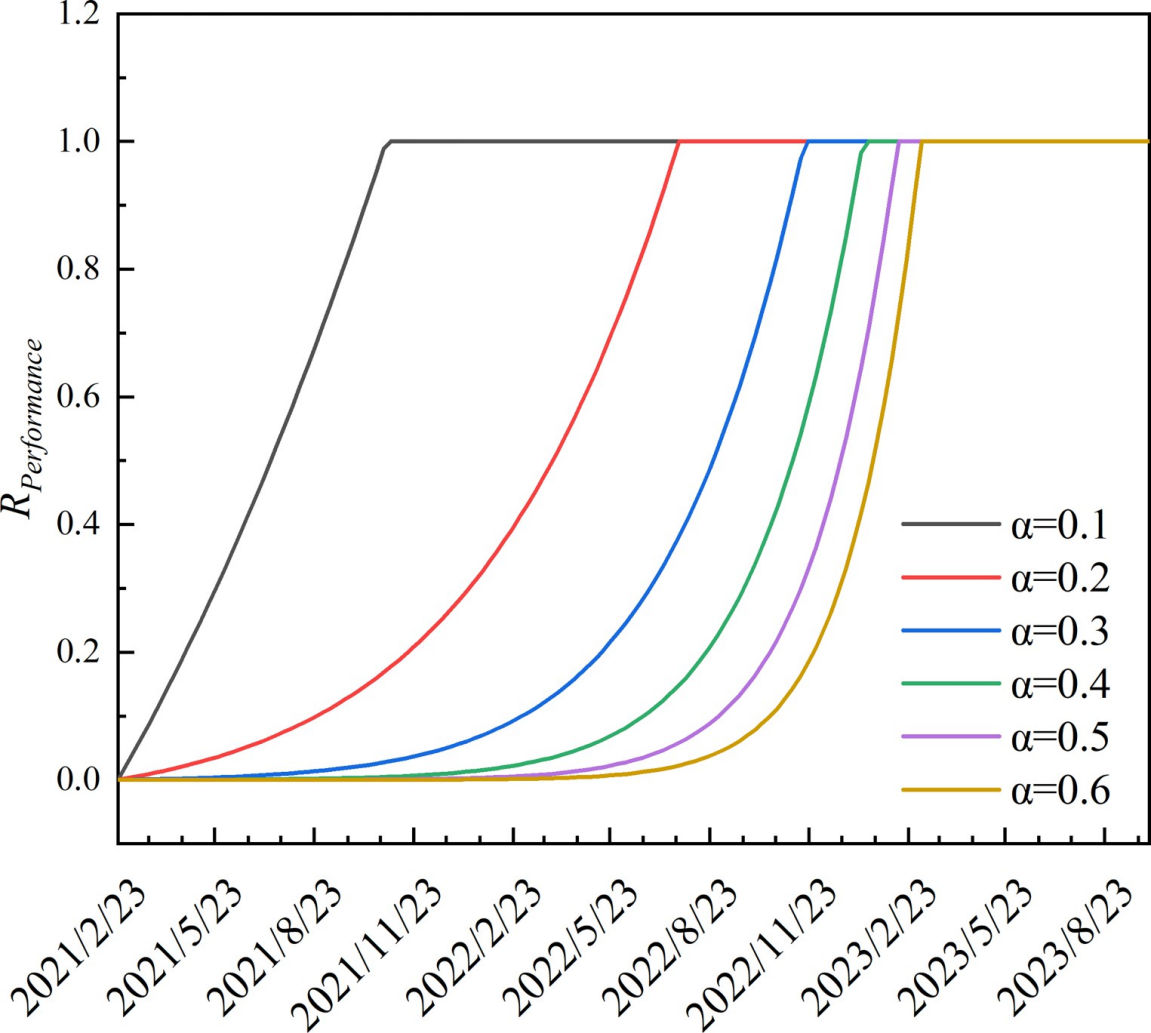
CRA function of airport network recovery pattern.

In the sigmoid recovery model, different types of recovery modes can be obtained by changing the composite recovery indices (*α*) [38]. When *α*<0.3, the network is more responsive, and recovery time is rapid. When *α*>0.3, the network is less responsive, but recovery is more rapid in later periods. To prevent both gradient disappearance and explosion in the recovery function, we specify *α*∈[0.2,0.6] (Fig 2).

The composite recovery index $\left(\alpha^{A_{m}}\right)$ combines the relative loss of system performance $\left(\beta_{L}^{A_{m}}\right)$ and different strengths of epidemic prevention and control strategies $\left(\gamma^{A_{m}}\right)$, which is defined in Eq 5:

$$\alpha^{A_{m}}=\beta_{L}^{A_{m}}\gamma^{A_{m}}$$ (5)

where $A_{m}=\{CAN,EAN,AAN\}$ are the different region airport networks.

$\beta_{L}^{A_{m}}$ is a metric used to evaluate the relative loss of airport network performance, as shown in Eq 6.


$$\beta_{L}^{A_{m}}=\frac{R_{loss}}{R_{performance}}$$ (6)

Although this study focuses on the impact of epidemic prevention and control strategies on airport networks, $\gamma^{A_{m}}$ represents the stringency of prevention and control policies in a certain period, as shown in Eq 7.

$$\gamma^{A_{m}}=\frac{\bar{SI}}{100}$$ (7)

where $\bar{SI}$ is the average stringency index of the prevention and control policy responses in each country/region.

The performance recovery of the airport network at a specific period is calculated using Eqs 4–7 which is defined in Eq 8.


$$R_{t}=\int_{0}^{t}R_{re\mathrm{cov}ery}$$ (8)

Notes: Eq 6 will no longer predict the evolutionary performance level of the airport network resilience system after the system has been restored to its pre-disruption performance.

#### 3.2.3. Resilience measure index

Nan and Sansavini (2017) used a quantitative approach to assess the resilience of infrastructure and proposed a resilience evaluation model, *GR*, which considered the three capacities of absorption, adaptation and recovery, and different recovery stages [39]. Wang and Miao (2020) constructed an airspace sector resilience model, and used *GR* to assess the resilience level of the airspace sectors [40]. Guo et al. (2021) assessed four characteristics of airport network resilience when subjected to public health events: robustness, redundancy, rapidity, and learning [20].

Robustness (*R*) represents the ability of a network to maintain its basic stability after a disturbance, and is defined by Eq 9.

$$R=\min\{R_{Performance}(t)\}$$ (9)

where *R*_*Performance*_(*t*) is network performance at *t* time.

The performance loss in the disruptive phase (*RAPI*_*DP*_) measures the speed at which the network loses performance after disruption, which is defined by Eq 10:

$$RAPI_{DP}=\frac{R_{damage}}{t_{r}-t_{d}}$$ (10)

where *t*_*r*_ is the network recovery start time and *t*_*d*_ is the time of the disturbance.

Rapidity (*RAPI*_*RP*_) measures how promptly the network recovers after a disruption, and is defined in Eq 11.

$$RAPI_{RP}=\frac{R_{t}}{t_{ns}-t_{r}}$$ (11)

where *t*_*ns*_ is the time needed for recovery to initial performance.

The time-averaged performance loss (*TAPL*) is the performance loss of the system under disturbance, and is defined by Eq 12.

$$TAPL=\frac{\int_{t_{d}}^{t_{r}}(R_{Performance}(t_{0})-R_{Performance}(t))dt}{t_{r}-t_{d}}$$ (12)

where *R*_*Performance*_(*t*_0_) is the network’s initial performance.

The recovery ability (*RA*) evaluates the performance of the network in reaching the stabilization phase, and is defined in Eq 13.


$$RA=|\frac{R_{t}}{R_{damage}}|$$ (13)

The resilience metric (*G*_*RE*_) comprehensively measures the entire process before and after the network is subjected to external disturbances, and is defined in Eq 14.


$$\begin{array}{l} G_{RE}=f(R,RAPI_{DP},RAPI_{RP},TAPL,RA)\\ =R\times \left(\frac{RAPI_{DP}}{RAPI_{RP}}\right)\times {\left(TAPL\right)}^{-1}\times RA \end{array}$$ (14)

## 4. Experimental analysis and discussion

In this section, we evaluate the performance and resilience of different airport networks.

### 4.1. COVID-19 impact analysis

**Fig 3:**
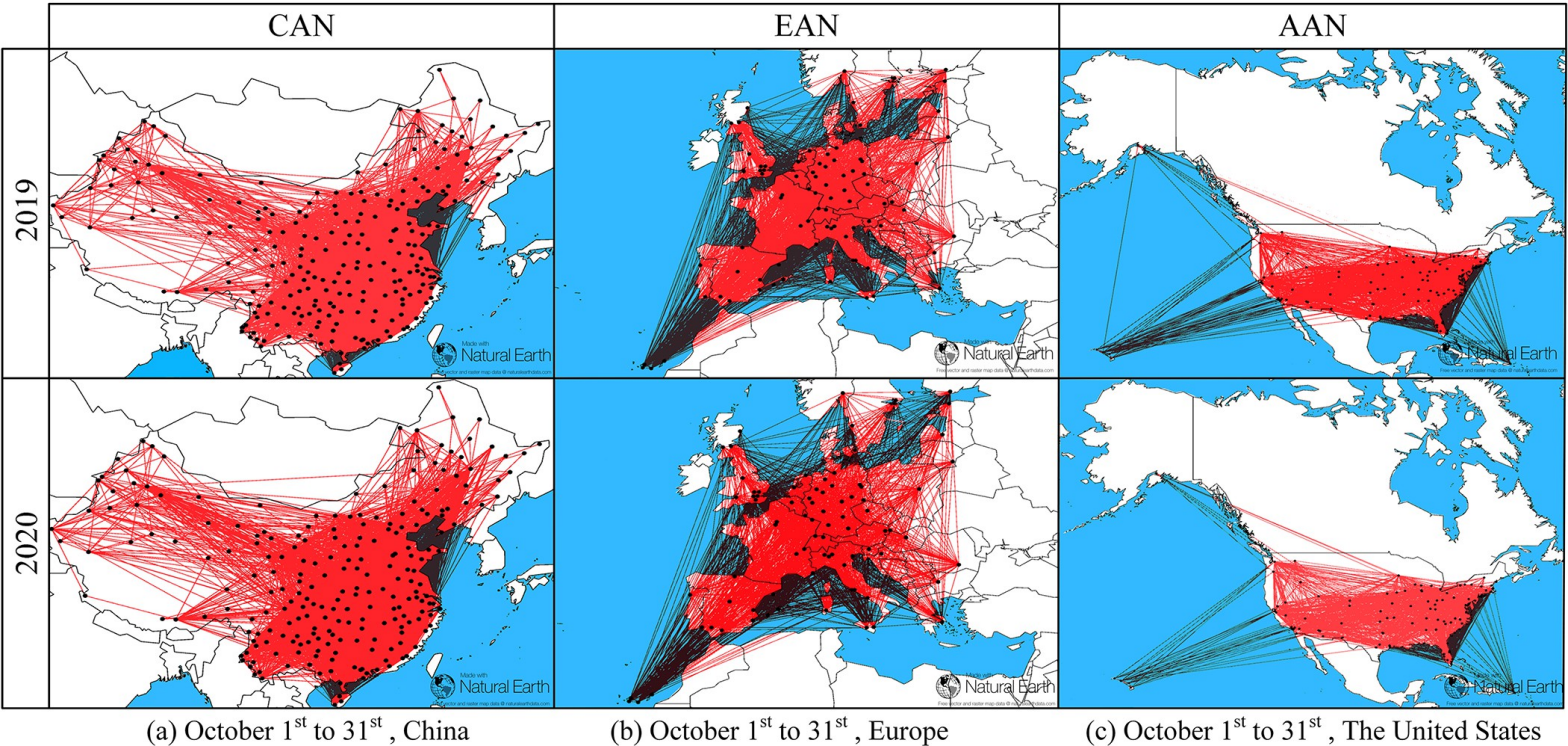
CAN, EAN and AAN weighted airport topologies for the period October 1 to 31 before and during the COVID-19 pandemic.

Fig 3 shows the weighted airport network topologies for CAN, EAN and AAN six months after the most serious COVID-19 pandemic outbreak (from April 2020).

The degree of airport connectivity of CAN in 2020 is slightly sparser than that in 2019, with northwestern regions, such as Xinjiang having significantly lower connectivity with other regions. The CAN topology structure indicated that most small- and medium-sized cities were connected to other regions via large urban airports. In contrast, the EAN was significantly less connected during COVID-19, having closed 806 air routes (12.29% of the total routes). The ANN closed multiple routes that affected Alaska and Hawaii. However, during the epidemic, the EAN had a more intensive degree of connectivity than the CAN and AAN, while the AAN partial airport network mapped the United States of America airport network with high connectivity and excellent network transmission performance.

**Fig 4:**
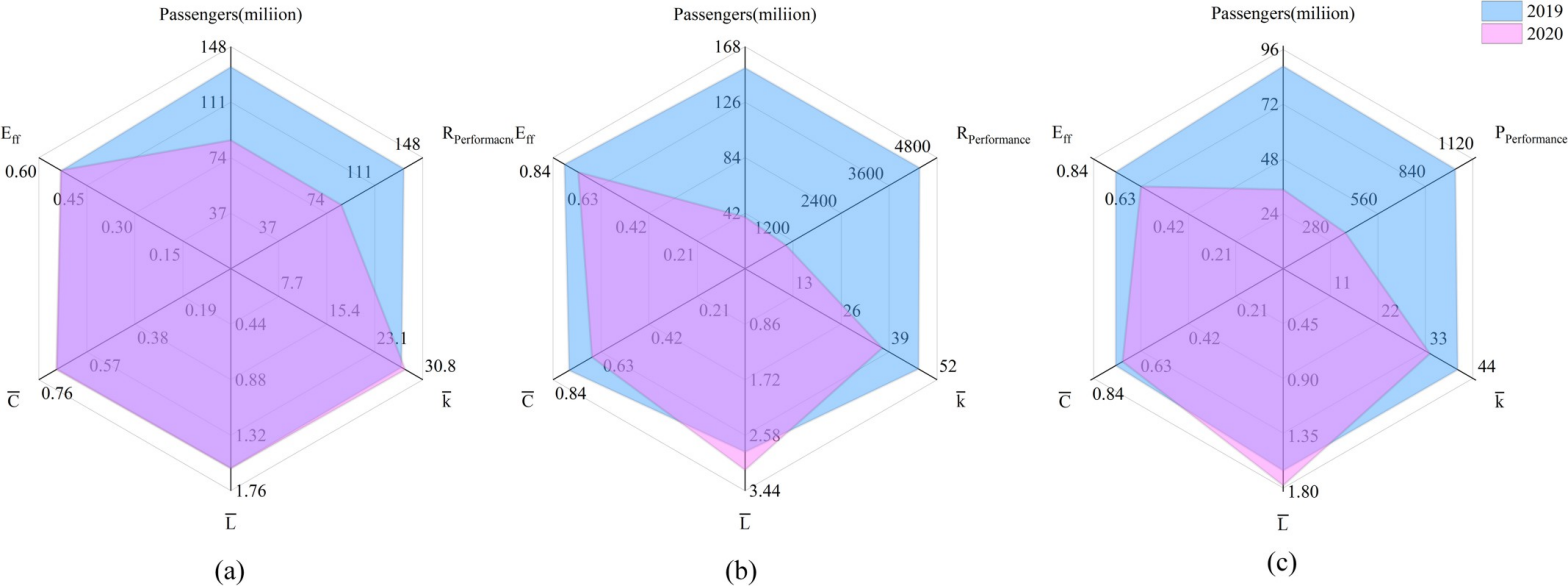
Topological indicators of changes to airport networks during COVID-19.

In Fig 4, we use $\bar{k}$, $\bar{C}$, $\bar{L}$, *E*_*ff*_, passenger throughput, and *R*_*Performance*_ to assess the change in the performance of the airport network.

The changes in the CAN are shown in Fig 4A. The CAN structure experienced minor damage; $\bar{L}$, $\bar{C}$, and *E*_*ff*_ were almost unchanged during the epidemic, and *R*_*Performance*_ of CAN decreased the least (36.35%) amongst the networks. However, in terms of its comparative robustness, CAN under-performed. The EAN structure experienced considerable disruption during the epidemic, as indicated by the significant changes in $\bar{k}$, $\bar{C}$, $\bar{L}$, and *E*_*ff*_ (refer to Fig 4B). The closure of many air routes in Europe implied that the *R*_*Performance*_ of EAN decreased by 74.24%, nearly four times the decrease in AAN, and 40 times that of CAN. Fig 4C shows the changes to AAN: $\bar{k}$ and *E*_*ff*_ increased by 15.99% and 14.6%, respectively, but $\bar{L}$ and $\bar{C}$ changed minimally. This indicates that the AAN structure was not disrupted significantly, although the degree of connectivity was reduced. Although AAN closed more air routes than EAN, the reduction in *R*_*Performance*_ in AAN was less than that in the EAN (60.94%), suggesting that the AAN has lower performance than the EAN.

### 4.2. Post-pandemic resilience analysis of airport networks

The ICAO predicted the post-pandemic recovery time for civil aviation as probably taking until 2024 [2]. Therefore, the maximum acceptable recovery time of the airport network resilience system was set at 48 months. Accordingly, we simulated and predicted the recovery of the airport network in each country/region and speculated the restoration time of the airport network in each area.

Eqs 5–7 allows one to obtain composite recovery indices for different networks (refer to Table 1).

**Table 1:** CRA composite indices by airport network under COVID-19.

	$\alpha^{A_{m}}$	$\beta_{L}^{A_{m}}$	$\gamma^{A_{m}}$
CAN	0.2694	0.3635	0.7412
EAN	0.4111	0.7424	0.5537
AAN	0.3347	0.6094	0.5492

**Fig 5:**
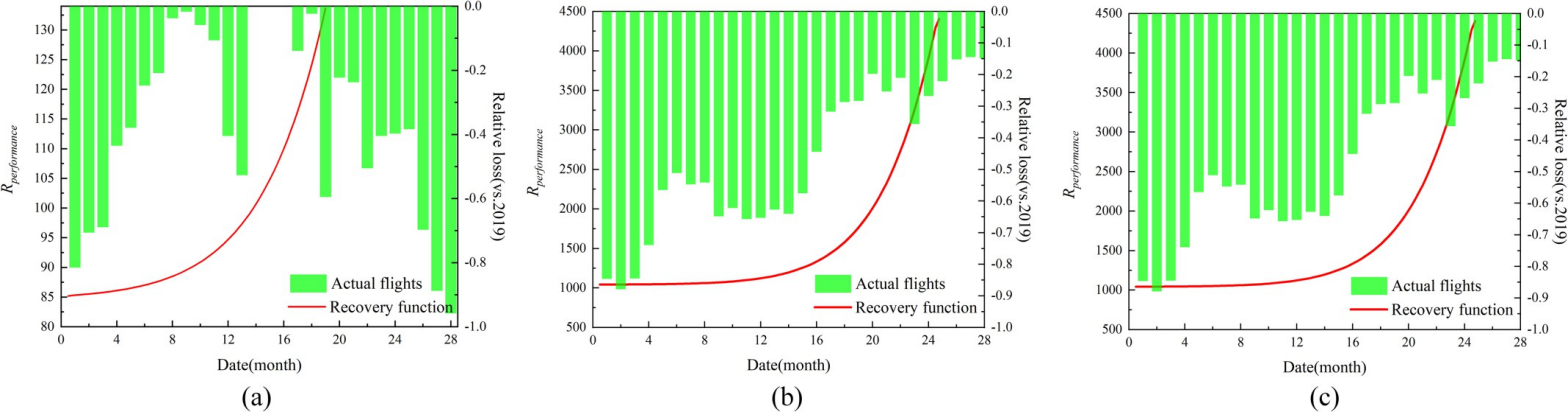
Recovery comparison of airport network.

We predicted the resilience of airport networks using the CRA model shown in Fig 5. Variations in epidemic prevention and control measures have caused variations in the recovery rate. In the CRA prediction, the CAN recovery time was the shortest, and the AAN recovery time was the longest. In terms of the recovery function, the smaller the value of $\alpha^{A_{m}}$, the faster the response.

Fig 5A shows the CAN recovery variation and predicted recovery time of 19 months (August 2021) in CRA. The stringency policy is to recover the CAN to 2019 status in a relatively short time. Flights of CAN recovery reached 5.5% in May (vs.2019). However, the continued stringency policy has not kept CAN recovering and led to a sudden increase and decrease in the actual flights. This illustrates that the adoption of stringency policies such as travel restrictions and household can maximize the recovery of airport network performance in a short period, but continuous stringency was not beneficial to the recovery and operation of the airport network. In particular, the crash of China Eastern airline in March 2022 led to a sudden drop in the number of flights (relative loss of 88.82%). This accident and COVID-19 happened simultaneously, which seriously affected the operation of Chinese civil aviation.

Fig 5B and 5C show the EAN and AAN recovery variation, and predicted recovery time of 25 months (April 2022), and 26 months (July 2022) in CRA. Flights of EAN recovery reached -15.2% in May (vs.2019) and AAN recovery reached -8.1% in July (vs.2019). The policies in EAN and the AAN were changed in the early period, and the flight volume gradually recovered to 2019 status. There was no sudden reduction in flight volume again, which shows that the policies adopted by Europe and the United States were beneficial to the recovery and operation of the airport network.

**Fig 6:**
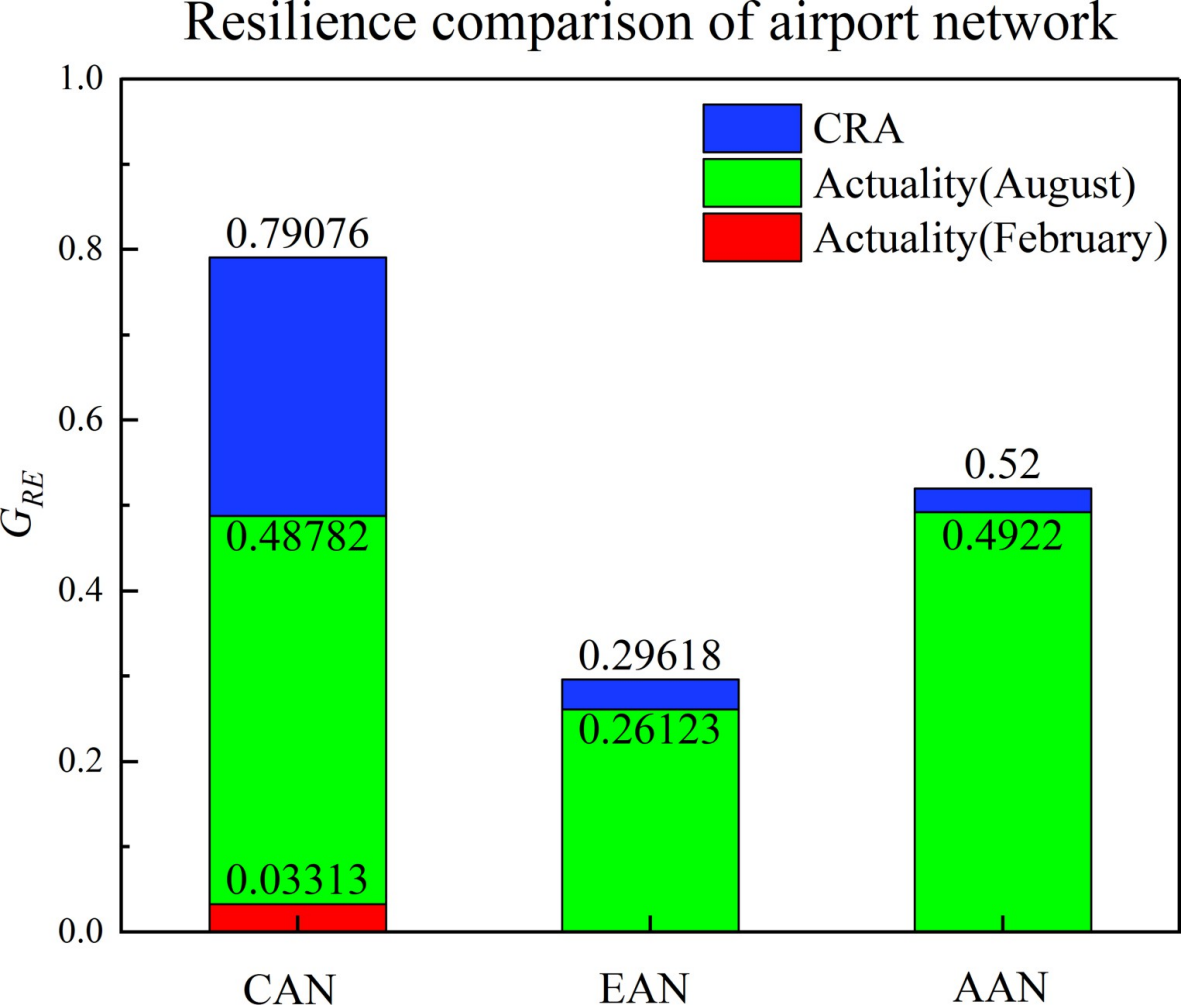
Resilience comparison of airport network.

Eqs 8–14 are used to calculate the level of airport network resilience, *G*_*RE*_ for each airport network given their differing epidemic prevention and control strategies (refer to Fig 6). The CAN resilience was better because it has the lowest network performance loss and shortest predicted recovery time. However, due to stringency policies and crash events, resilience was significantly affected. In particular, the resilience of the CAN caused by the crash is almost zero, indicating that the safe operation of civil aviation will directly affect the resilience and performance of the airport network. The EAN and AAN resilience was marginally different between the actual and predicted, which indicates that the policies adopted by the EAN and AAN are beneficial to maintaining the resilience of the airport network.

## 5. Conclusions

In this study, we analyzed the correlation between airport network resilience and epidemic prevention and control policy responses in the three most prolific aviation countries/regions: China, Europe, and the USA. We used network science and data-driven analysis as key tools and proposed a quantitative analysis methodology to model the resilience and recovery of airport networks based on the CRA model. This considers multiple influencing factors: loss of network performance and different stringency policies. The topological characteristics of the three airport networks changed between 2019 and 2020. From the domestic passenger throughput of the country/region (i.e., without international travel) perspective, higher stringency policy has less network performance loss and faster recovery rate in the short term. However, this limits the recovery rates for a long time.

In the early period of future global public health emergencies, countries/regions should jointly implement higher-stringency policies for prevention and control measures. CRA was used to model the recovery function of each network and predict its recovery time. The three networks use different stringency policies in their recovery strategies, reflecting their national conditions. Different recovery measures in the late recovery period do not significantly affect the recovery rate; however, prolonged and strict travel bans and other policies will significantly limit the recovery rate of the aviation industry. In addition, a widespread epidemic causes people to panic, which is detrimental to the recovery of the aviation industry. Different governments have adopted different policy responses based on different national conditions, such as population resources and health resource occupancy, which showed different degrees of recovery in the aviation network. Therefore, countries/regions should jointly discuss when to lift or reduce prevention control measures, build a vaccine passport system of mutual trust, not impose quarantines on countries/regions that have been free of the epidemic over a long period, and accelerate the recovery of international routes. This is conducive to the rapid recovery of the aviation industry. Ideally, national and regional health and epidemic prevention departments should cooperate to reduce the impact of COVID-19. In future, we will continue to study the influence of multiple disturbances on network resilience. Analyzing such multiple disturbance effects can help better respond to future global public health events.

## Supporting information

S1 File (ZIP)
